# *In situ* Observation of Phase Transformation in MnAl(C) Magnetic Materials

**DOI:** 10.3390/ma10091016

**Published:** 2017-08-31

**Authors:** Ping-Zhan Si, Hui-Dong Qian, Chul-Jin Choi, Jihoon Park, Sangho Han, Hong-Liang Ge, Kiran P. Shinde

**Affiliations:** 1 Powder & Ceramic Division, Korea Institute of Materials Science, Changwon, Gyeongnam 51508, R. Korea; qianhuidong@kims.re.kr (H.-D.Q.); jpark@kims.re.kr (J.P.); hsh6188@kims.re.kr (S.H.); kiranshinde@kims.re.kr (K.P.S.); 2 College of Materials Science and Engineering, China Jiliang University, Hangzhou 310018, China; hongliang_ge@cjlu.edu.cn

**Keywords:** phase transformation, MnAl, Mn_54_Al_46_, MnAlC, diffusion, magnetic properties

## Abstract

The phase transformation in two modes, including both displacive and massive growth of τ-phase from ε-MnAl(C), was observed by *in situ* transmission electron microscopy. The exact temperature range for different phase transformation modes was determined by magnetic measurements. The displacive growth of ε→τ in Mn_54_Al_46_ (or Mn_54_Al_46_C_2.44_) occurs at temperatures below 650 K (or 766 K), above which both modes coexist. One-third or less of the ε-phase can be transformed into τ-phase via displacive mode while the remaining two-thirds or more via massive mode. In bulk τ-phase, most τ-nanocrystals formed via displacive mode are distributed in the matrix of large τ-grains that formed via massive mode. The typical massive growth rate of the τ-phase is 8–60 nm/s, while the displacive growth rate is low. A more complete understanding of the ε→τ phase transformations in the MnAl-based magnets was provided in this work, based on which the annealing process for ε→τ was optimized and thus high purity τ-phase with high saturation magnetization was obtained.

## 1. Introduction

The L1_0_ structured τ-phase MnAl, usually prepared by annealing the hexagonal ε-phase MnAl at moderate temperatures, is attracting increasing research interests over decades for its low cost and high performance as promising rare earth free magnets [1,2,3,4,5,6,7,8,9,10,11,12,13,14]. The metastable nature of τ-MnAl usually results in its decomposition during prolonged annealing or high temperature processing [3,4,5,6]. The doping of C to MnAl can improve the stability of τ-MnAl for the reason that the interstitial C atoms inhibit the diffusion of Mn and Al atoms but quantitative studies on the relationship between thermal stability, carbon content, and phase transformation are still very rare [3,4,7,15]. The thermal-driven diffusion process has substantial effect on the ε→τ phase transformation (hereafter denoted by PT) in MnAl-based magnets. In this work, we systematically studied the PT mechanism in MnAl(C) alloys by several methods. Full understanding of the ε→τ phase transformation is important for the preparation of high purity τ-phase and the development of high performance MnAl–based magnets.

The phase transformation of ε→τ in MnAl has been studied by several groups over decades [16,17,18,19,20]. Early X-ray diffraction (XRD) studies showed that the hexagonal-close-packed ε-phase transforms into an orthorhombic (ε’) phase by an ordering reaction first, and then to the metastable ferromagnetic face-centered-tetragonal τ-phase by a diffusionless displacive shear reaction [12]. According to Broek, the displacive mode of the ε→τ PT follows the sequence ε→ε’→polytypes→τ, where the τ-phase was assumed to nucleate in ε’-domain [17]. The later metallographic studies indicated that the ε→τ occurs via a compositionally invariant, diffusional transformation akin to the so-called massive transformation rather than a displacive or martensitic transformation [18]. The coexistence of the diffusional massive mode and the displacive shear mode during the formation of τ-MnAl was also reported [16]. A hybrid displacive-diffusional mechanism involving the motion of partial dislocations that act as transformation dislocations and concomitant short-range diffusion has been observed in recent years [19]. Different PT mechanisms have been observed in previous studies based on different samples, but the relationship between these mechanisms and the factors that trigger different mechanisms were not clear due to the following reasons.

Most previous transmission electron microscopy (TEM) observations on the ε→τ were carried out on postmortem samples, in which the ε- and τ-phases were formed prior to the room temperature examinations by TEM [16,17,18,20]. The details of the ε→τ PT occurred at high temperatures before observation was restored indirectly through reasoning and imagination. However, it is difficult to imagine the formation sequence of different τ-phase grains observed at the same time, leaving losing or incomplete details of the process. Wiezorek et al. reported some details of the dynamic sequences of PT during the *in situ* TEM heating experiments conducted at temperatures between 813 K and 923 K, at which both the dominating massive ordering mode and the hybrid displacive-diffusional mode were observed [19]. The displacive mode usually occurs at lower temperatures while the massive mode dominates at high temperatures, but the exact temperature range is unknown.

Since the rate for ε→τ massive transformation is so fast that it usually completes within several tens of seconds to typically no more than 20 min, it is important to select proper time windows to capture the dynamic details by *in situ* TEM before the completion of the PT. A higher temperature may result in very fast PT so that we do not have enough time for observation. A lower temperature may result in partial transformation by merely displacive mode rather than massive mode. In this work, we determined the onset temperature of displacive mode and massive mode by using magnetic measurement, based on which the temperature with a medium rate of ε→τ was selected for *in situ* TEM to make sure that we have enough time to capture more details of the transformation.

## 2. Materials and Methods

The alloys with nominal composition of Mn_54_Al_46_ and Mn_54_Al_46_C_2.44_, hereafter denoted by MnAl and MnAlC, were produced from manganese (purity 99.95%), aluminum (purity 99.999%) pieces, and high purity carbon granules with a high-frequency vacuum induction furnace in argon atmosphere (purity 99.999%). The melt was cast into a bar (φ = 13 mm) using an alumina mold with subsequent natural cooling in vacuum. The compositional deviation due to the volatility of Mn in the molten state was minimized by maintaining the molten state within 3 min. To increase the homogeneity, the as-cast alloys were annealed for 24 h at 1423 K (at which the high temperature ε-phase is stable), and then quenched into water to prevent the decomposition of the ε-phase into the equilibrium γ_2_ and β-phases, and the formation of the τ-phase. After that, the ε-phase was annealed at 773 K for varied time intervals to produce τ-phase. The heating/cooling rate during annealing is 20 K/min.

The XRD patterns were collected at room temperature by a Rigaku D/Max 2500 automatic diffractometer operating at 40 kV and 100 mA, in θ–2θ configuration, with Cu-K_α_ radiation (Ni filter, λ = 1.5418 Å). A fixed divergence and anti-scattering slit giving a constant volume of sample illumination were used. The angular step in 2θ is 0.02°. Phase identification was evaluated using the Powder Diffraction File database. The PT of the ε-Mn_54_Al_46_C_2.44_ at 773 K was *in situ* heated at 20 K/min and was studied by using a JEOL 200CX TEM. Thin foils of the as-quenched ε-phase for TEM observations were prepared by using a focused ion beam workstation.

The magnetic properties were measured using a Quantum Design physical property measurement system. The temperature dependence of magnetization of the ε-MnAl and ε-MnAlC was measured with increasing temperature (20 K/min) under an applied field of 2 Tesla. The paramagnetic ε-phase transforms into τ-phase at temperatures above its Curie point, above which the τ-phase is paramagnetic and thus a magnetic field of 2 T is applied to probe the magnetization changes of the paramagnetic ε-phase to the paramagnetic τ-phase at high temperatures. The ε-MnAlC was annealed under 4 T for 13 h at 623 K and 773 K, respectively. The magnetic hysteresis loops of the annealed samples were measured at room temperature. The time dependence of magnetization of ε-MnAl at 4 T was recorded at 573 K and 673 K, respectively.

## 3. Results and Discussion

### 3.1. Phase Transformation Probed by XRD

**Figure 1:**
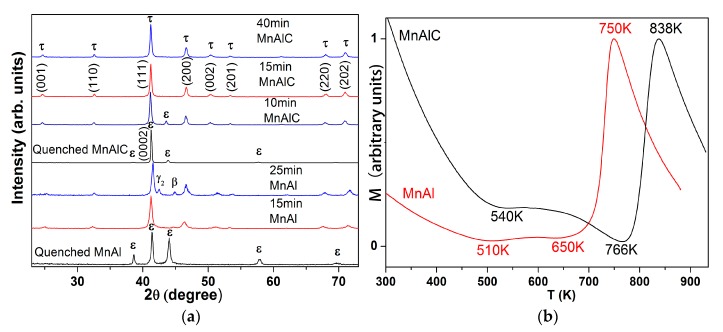
(**a**) XRD patterns show that the water-quenched MnAl and MnAlC are single ε-phase. Single τ-phase was obtained in MnAl annealed for 15 min and in MnAlC annealed for 15 and 40 min. A small fraction of τ-phase in MnAl decomposed to γ_2__-_ and β-phase when annealed for 25 min. The MnAlC annealed for 10 min is composed of ε- and τ-phases; (**b**) Normalized M-T curves of ε-MnAl/ε-MnAlC under 2 T with increasing temperature.

The XRD patterns of both MnAl and MnAlC alloys after 1423 K-homogenization followed by water quenching could be indexed with a single ε-phase, as shown in Figure 1a. Both ε-MnAl and ε-MnAlC, when heated at 773 K, transformed into pure τ-phase in 15 min. Trace amounts of γ_2_ and β phases precipitate from τ-MnAl after heating for 25 min, indicating decomposition of τ-MnAl under prolonged heating time at 773 K, owing to the lower decomposition temperature of τ-MnAl as determined by magnetic measurements below. However, no decomposition was found in the τ-MnAlC after heating for 40 min, indicating a structural stabilization effect of carbon in the lattices. Both ε- and τ-phases exist in MnAlC heated for 10 min, indicating incomplete PT in this stage. The time windows for the precipitation of different phases during the ε→τ PT at 773 K are important for guiding the following *in situ* TEM heating and observations. The stress level and grain size of the ε- and τ-phase, both of which are metastable, might have effect on the position and broadening of the XRD peaks. The (111) peak of the τ-MnAl obtained by 25-min annealing shifts slightly to a higher degree in comparison with that of the τ-MnAl obtained by 15-min annealing, indicating smaller lattice parameters of τ-MnAl after prolonged annealing. The reduced lattice parameters of the τ-MnAl obtained by 25-min annealing were ascribed to its partial decomposition during prolonged annealing that can, to some extent, release the lattice stresses. In comparison with τ-MnAlC, the τ-MnAl exhibits broadened diffraction peaks, as shown in Figure 1a, indicating smaller grain size of τ-MnAl than that of τ-MnAlC.

It is interesting that the ε-MnAlC shows a much stronger diffraction peak of (0002) planes and very weak diffraction of the other planes. For comparison, the X-ray diffraction intensities of different planes in ε-MnAl do not vary much. According to the Scherrer equation, the intensity of X-ray diffraction is largely dependent on the crystalline dimensions or size in the samples. Therefore, the strong diffraction peak of the (0002) plane in ε-MnAlC might indicate a larger dimension of the ε-phase along the c-axis than that along directions perpendicular to the c-axis. The comparable diffraction intensities of different peaks in ε-MnAl indicate comparable dimensions of the ε-phase in different directions. It seems that the carbon atoms in ε-MnAl(C) lattices hinder the growth of ε-phase along the basal plane and thus the dimension of atomic long-range ordering along the basal plane is reduced.

### 3.2. Phase Transformation Probed by M-T Curves

Figure 1b plots the temperature dependence of magnetization of the ε-MnAl and ε-MnAlC measured with increasing temperature (20 K/min) under an applied field of 2 Tesla. The magnetization of paramagnetic ε-MnAl/ε-MnAlC decreases with increasing temperature from 300 K due to thermal agitation. However, the downward tendency of M reversed at temperatures above 510 K and 540 K for MnAl and MnAlC, respectively, indicating transformation of paramagnetic ε/ε’ to ferromagnetic τ-phase. The magnetizations of MnAl and MnAlC do not vary too much at 510–650 K and 540–766 K, respectively, indicating the occurrence of additional magnetization in compensation of the thermal driven magnetization loss in this temperature range. The PT during this stage was slow and was ascribed to the displacive PT, which is a low temperature diffusionless process involving co-operative shear movements of atoms on (001) ε’ along [010] ε’ that produces the final tetragonal lattice of τ-phase.

A sharp magnetization increase was observed at temperatures above 650 K and 766 K for MnAl and MnAlC, respectively, indicating occurrence of high-rate PT in the samples. The PT during this stage was fast and was ascribed to the massive growth of τ-phase from paramagnetic ε/ε’-phase. It should be noted that the τ-phase is paramagnetic in this temperature range for the Curie temperature *T_c_* of our τ-MnAl and τ-MnAlC is measured to be ~630 K and ~550 K, respectively. This sharp magnetization increase for τ-phase in high temperature paramagnetic state could be explained by Curie–Weiss law. The M of most substances, no matter ferromagnetic or paramagnetic, normally decreases with increasing temperature in the presence of a certain external field due to thermal agitation. However, for substances with thermal driven phase transformation, the fraction change of different phases may result in abnormal temperature dependence of magnetization. For ferromagnetic τ-phase at temperatures above *T_c_*, the paramagnetic behavior follows the Curie–Weiss law, *M* = *CH/(T −* θ*)*, where the term θ describes the exchange interaction that is present albeit overcome by thermal motion. The sign of θ is positive for ferromagnetic τ-phase and is negative for antiferromagnetic/papramagnetic ε-ε’-phase, respectively. As a result, the paramagnetic τ-phase shows much larger magnetization than ε-phase.

At temperatures above 750 K (838 K), the magnetization of MnAl (MnAlC) decreases quickly with increasing temperature, indicating decomposition of the ferromagnetic τ-phase or possibly a completion of the PT. This result shows the reason for the decomposition of τ-MnAl and no decomposition of τ-MnAlC at 773 K as observed in the above XRD patterns. The annealing temperature of 773 K is higher (lower) than the decomposition temperature of τ-MnAl (τ-MnAlC). As a result, τ-MnAl decomposed while τ-MnAlC did not decompose after prolonged heating time.

It is known that the addition of a small amount of carbon (within the solubility limits) could stabilize the τ-phase of MnAl as evidenced that τ-MnAl tends to decompose into γ_2_ and β-phase while τ-MnAlC does not decompose in the same heat treatment conditions. The mechanisms for such a stabilizing effect can be found in Figure 1b, which shows that the minimal temperature (766 K) for activation of massive growth of τ-MnAlC is higher than the decomposing temperature (750 K) of τ-MnAl. As a result, it is not strange to observe the decomposition of τ-MnAl if we want to obtain τ-MnAlC by the same heating process. In fact, carbon not only increases the decomposition temperature of τ-phase, but also increases the activation temperatures for both displacive and massive transformations of ε-phase, as shown in Figure 1b. This result is in agreement with the results in reference [4]. It seems that carbon increased the energy barriers of the PT and phase decomposition.

### 3.3. Short-Range Displacive Mode Probed by In situ TEM

**Figure 2:**
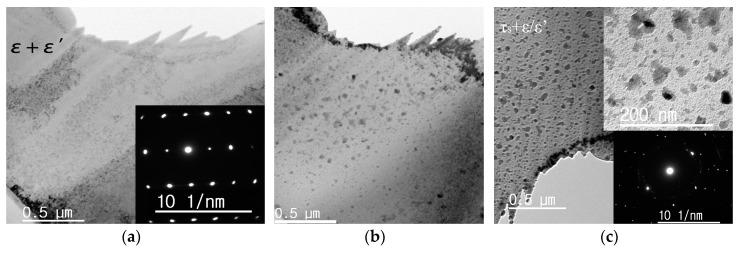
*In situ* TEM images of MnAlC at 773 K. (**a**) The parent ε/ε’ phase shows continuous bright contrast; (**b**) With increasing time, the τ-phase, shown as black dots distributed in the ε/ε’ matrix, was formed via displacive mode; (**c**) The density and size of the τ-grains formed via displacive mode (τ_s_) increase with time. An enlarged view as shown in the inset of (**c**) shows that the τ_s_ grains grow up to 40 nm slowly and cover approximately one-third of the field in view. The electron diffraction patterns of the samples are shown in the inset of (**a**,**c**), respectively.

The *in situ* heating TEM bright field images of the ε-MnAlC within the initial 10 min are shown in Figure 2. The parent ε-MnAlC shows a continuous bright contrast before heating, as seen in Figure 2a. It had been proved that the water-quenched materials, which from X-ray analysis were expected to be pure hexagonal ε-phase, already contain numerous small nuclei of the ordered orthorhombic ε’-phase that are 4–10 nm in size, which grows to 30–40 nm upon heating to 723 K [17].

The ε→ε’ process is one of ordering in the close-packed *c*-plane of the six-fold symmetry hcp structure. The reduction in symmetry to twofold caused by the ordering results in the orthorhombic unit cell of the ε’-phase. Therefore, there are three symmetry-equivalent variants for ε’ in one original ε crystal. The orientation relationships between the ε-phase and the three variants of ε’ precipitates are as follows: [19]

ε_1_’: (0001) ε//(001) ε_1_’ and [11 20] ε//[010] ε_1_’

ε_2_’: (0001) ε//(001) ε_2_’ and [1 210] ε//[010] ε_2_’

ε_3_’: (0001) ε//(001) ε_3_’ and [ 2110] ε//[010] ε_3_’

The ε/ε’ phase, when heated for several minutes, starts to precipitate small τ-grains, as shown in Figure 2b. Figure 2c shows that the small τ-grains embedded in the ε/ε’ matrix grows slowly to a maximum size of about 40 nm with increasing heating time. It is interesting that these evenly distributed τ-grains could not grow further when its size reached ~40 nm, which is the size of a ε’ grain as observed previously [17]. The distribution of isolated island-like τ-grains embedded in ε-matrix is quite different from the massive transformation characteristics, but could be well explained by the displacive mode.

Although there are three variants of ε’, only one type of partial dislocation can shear one of the three possible ε’-phase variants that may be produced in a given ε-grain into the L1_0_ structure with the required correct ordering of atoms. If several partial dislocations glide on every other close-packed plane of the parent phase in which there are two or three variants of ε’, the resulting structure will not be L1_0_. Hence, in a given ε-phase grain, one ‘correct’ variant and two obstacle variants of the ε’-phase exist [19]. The two obstacle ε’ variants neighboring to the correct one have largely restrained the maximal size of τ-grain formed by displacive mode. The τ-phase grows through the motion of the partial dislocations along the close-packed plane. When a dislocation encounters the obstacle variants of ε_2_’ and ε_3_’, its motion is obstructed. The ε_2_’ and ε_3_’ variants have to reorder to become τ-phase. However, motion of a partial dislocation group with a Burgers vector that transforms ε_1_’ into the correct L1_0_ order would not lead to the formation of the L1_0_ structure from ε_2_’ and ε_3_’, but to a higher energy structure which was not observed. The strain originating from the transformation of ε_1_’ to τ and obstacle effect of ε_2_’ and ε_3_’ would hinder the subsequent growth of τ-grains. There are also some larger τ-islands that were actually composed of two or three connected smaller τ-grains, owing to the presence of neighboring ε’ variants of the same type. The inset of Figure 2c shows that the region for τ-phase with dark contrast accounts roughly one-third of the total area in view. This further proved that only one of the three variants was transformed into τ-phase by displacive mode. More evidence for this one-third phenomenon could be found in the following magnetic measurements. The small τ-gains are also difficult to propagate to the neighboring ε’ grains through the boundaries via shear mode due to incoherent interfaces and increasing shear strains.

In fact, it is difficult to observe the transformation sequence of these three ε’ variants by postmortem TEM because we could not distinguish the τ-phase formed by different variants. Our *in situ* TEM observations provide the time resolution for these transformations. Besides many small τ-grains formed in the parent ε-grains through shear mode (marked as τ_s_), a much larger τ-grain with size up to 180 nm nucleated at grain boundary has also been observed with increasing observation time. The τ-phase grown at grain boundaries has been proved to be crucial for the massive mode of PT [19,20].

### 3.4. Long-Range Massive Mode Probed by In situ TEM

The massive transformation is generally defined as a compositionally invariant nucleation and growth process involving a change in crystal structure and/or degree of long-range order. The growth of the massive τ-phase in MnAl is accomplished with the migration of incoherent heterophase interfaces by essentially random atomic attachment across the growth interface and is associated with the genesis of characteristic defect such as stacking faults, microtwins, and antiphase boundaries in the τ-phase product [19]. Since the shear mode itself could not transform all the ε-phase into τ-phase, a diffusion controlled reordering process must occur for all the three ε’ variants to transform into τ finally, but it is not clear when it takes place. Two possible mechanisms for (ε_2_’ and ε_3_’)→τ were proposed in the previous work [16]. It might occur in the bulk as a consequence of coarsening of ε_1_’. A second possibility is that the reordering occurs near the core of the transformation dislocations where diffusion is enhanced. Our work shows that the ε_2_’ and ε_3_’ transformed into τ mainly through thermally activated massive diffusional process. As mentioned above, the grain size of τ formed via coarsening of ε_1_’ usually is no larger than 40 nm, thus the enhancing diffusion in the ε_1_’→τ transformation dislocations should be very limited. For comparison, the growth rate and the maximal grain size of the τ-phase produced via diffusional process is much higher and larger than that formed via displacive mode.

**Figure 3:**
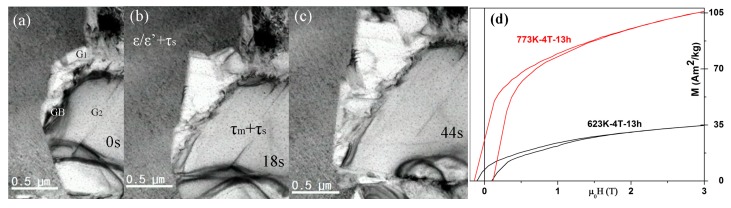
Massive growth of τ-phase. (From Video 1) (**a**) One τ-grain G_1_ hinders the growth of another τ-grain G_2_; (**b**) The τ_s_-grains (formed via displacive mode) were embedded in the matrix of growing τ_m_-grains formed via massive mode; (**c**) The growth rate of τ_m_ is estimated based on the size of the grains and the time required reaching it; (**d**) Room temperature M-H plots of the ε-MnAlC after 13-h annealing in 4 T.

Figure 3a–c (Video 1 in the supplementary materials) shows the growth process of the τ-phase via massive mode. These videos and micrographs captured a number of unique features of τ-phase formation in the MnAlC alloys. First, the small τ_s_-grains formed via displacive transformation from ε_1_’ make almost no change during the massive transformation of the surrounding ε/ε’ to τ_m_, resulting in a structure of τ_s_ embedded in τ_m_, as seen in Figure 3a–c. It should be noted that the size of the τ_s_-grains might vary a little with massive transformation rate and temperature. Since the τ_s_-grains near the massive transformation frontiers do not have enough time for full coarsening of τ_s_ via displacive mode, the grain size of τ_s_ thus should be smaller than those far from the massive transformation frontiers, as proved by the smaller grain size of τ_s_ in Figure 3 than that in Figure 2. Of course, the obscure boundaries between τ_m_ and τ_s_ might also result in a seemingly smaller τ_s_ size. Usually, the thermally activated massive transformation is accelerated with increasing temperature, a fast enough massive transformation might transform all the samples into τ in several seconds and give little time for τ_s_ to grow and for us to observe by *in situ* TEM. It is also possible for the metastable τ_s_ to be consumed by τ_m_ through diffusional process at higher temperatures. The τ-phase can form via massive mode without prior ε→ε’ ordering at high enough temperatures [19]. The annealing at 773 K provides a medium massive transformation rate and thus both modes are observed *in situ*.

Second, the rate of the propagation of the massive transformation front ranges from 8 nm/s to 60 nm/s, depending on the front shape. The propagation rates of the inter-phase interface with arc-shape, straight shape, and sharp-angle tip of τ_m_ are ~8, ~10, and ~16 nm/s, respectively. The ε/ε’ phase in the sharp angle in-between two τ_m_ grains transforms at a rate up to 60 nm/s, which slows down quickly when the sharp angle becomes obtuse due to ε/ε’→τ transformations. It should be noted that the high rate area is very limited while most linear growth rates are falling in the range 8–16 nm/s. Yanar et al. analyzed the growth kinetics of the τ phase in the Mn-Al-C alloys using modified Burke–Turnbull equation and postmortem TEM, yielding a linear growth velocity of ~1 μm/s [20], which is much larger than what we observed by *in situ* TEM. Yanar et al. determined the growth rate by dividing the growth distance by the estimated time of growth, while the growth distance was calculated by averaging maximum size measured in five τ colonies [20]. We speculate that one τ colony in the postmortem TEM sample might contain several or more τ grains grown from different nuclei at the same time, thus the growth distance might be overestimated. In fact, our *in situ* TEM studies showed that there are many τ grains grown from many different τ nuclei distributed in the sample. If several τ grains from different nuclei met together and grew into one τ colony, it is difficult to distinguish them by postmortem TEM observations. By applying the modified Burke–Turnbull equation and the average growth rate observed by our *in situ* TEM, the activation energy for diffusional growth in MnAlC is estimated to be 182.5 kJ/mol, which falls in the range of that reported by Lu et.al but is higher than that estimated by Yanar et al. [20,21].

Third, new τ grains tend to grow along the boundaries of the as-formed τ_m_ grains. The growth of one τ_m_ is hindered when encountered another τ_m_–grain. Stacking faults during the transformation are usually accumulated in the grain boundaries between the τ_m_ grains. The prominent facets that appear in the growing τ phase colonies are incoherent interfaces with no systematic orientation relationship with the parent ε/ε’ phase.

The magnetizations of MnAlC after fully PT at different temperatures through different modes further proved that one-third of the ε-phase in maximal could transform into τ via displacive mode. Figure 3d shows the room temperature magnetic hysteresis loops of the ε-MnAlC after 13-h annealing in 4 T. The field-assisted long-time annealing provides full transformation of τ-phase in ε-MnAlC at 623 K via displacive mode and at 773 K via both modes. The saturation magnetization (35 Am^2^/kg) of the 623 K annealed sample is approximately one-third of that (~105 Am^2^/kg) in the 773 K annealed sample. This proved that only one of three possible ε’ variants were transformed via displacive mode into τ-phase at 623 K, a temperature at which massive transformation is impossible. This is in good agreement with the above TEM observations, in which τ_s_ grains covered approximately one-third of the field in view. The Curie temperature for our τ-MnAl and τ-MnAlC is measured (not shown here) to be 630 K and 550 K, respectively. The addition of carbon significantly decreased the Curie temperature of τ-MnAl.

### 3.5. Phase Transformation Fraction/Rate Probed by M-H/M-t Curves

**Figure 4:**
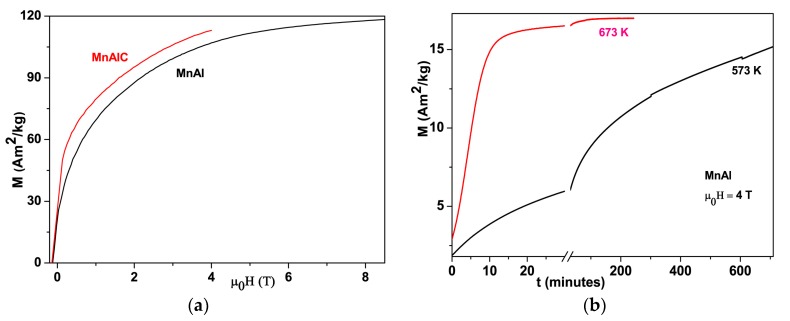
(**a**) Room temperature M–H curves of τ-MnAl and τ-MnAlC; (**b**) The M–t plots of ε-MnAl during field-heating at 573 K and 673 K, respectively.

The magnetization of the samples is largely dependent on the fraction of transformation from nonmagnetic ε-phase to ferromagnetic τ-phase. Figure 4a shows the demagnetization curves of τ-MnAl and τ-MnAlC at 300 K. The magnetization of our τ-MnAlC and τ-MnAl reached up to ~114 Am^2^/kg at 4 T, and 118.3 Am^2^/kg at 8.5 T, respectively. The room temperature *M_s_* of MnAl-based magnets prepared by the traditional two-step process, including melting and annealing steps, was reported to be ~73 Am^2^/kg at 2 T [22], ~82 Am^2^/kg at 2 T [10], 94 Am^2^/kg at 3 T [23], and 100 Am^2^/kg at 14 T [24]. The room temperature *M_s_* of samples prepared by one-step process, including one-step strip casting technique and direct drop synthesis method, has been reported recently to be 114 Am^2^/kg at 5 T [7], and 117 Am^2^/kg at 9 T [9], respectively. The magnetization of our samples prepared by the traditional two-step process is higher than most previously reported values, indicating higher fraction of the τ-phase in our samples and the effectiveness of our method in controlling the PT. An *M_s_* of ~128 Am^2^/kg was reported in the samples with Zr substitution for Mn and high C content samples with 72 wt. % Mn [13,25]. We also studied the effect of magnetic field during annealing on the phase transformation and found that the magnetic field has little effect on the saturation magnetization of the final product.

The time dependence of magnetizations of ε-MnAl at 573 K and 673 K are shown in Figure 4b, which to some extent shows the PT rate/fraction of ε→τ with time. The magnetization of ε-MnAl, when heated at 673 K, increases quickly within the first 15 min and then slowly until stabilizing at a certain value after 100 min, indicating full transformation of the ε- to τ- phase. However, no saturation was observed in ε-MnAl when heated at 573 K for more than 12 h, indicating very slow PT rate at this temperature. As mentioned above, for ε-MnAl, the displacive mode acts at 573 K while massive mode dominates the PT at 673 K. Fast transformation rates are the characteristics of the massive transformation. The low transformation rate of displacive mode is ascribed to the obstacle effect of ε_2_’ and ε_3_’ to the shearing process of ε_1_’ variants. In fact, the coarsening rate of τ_s_ is observed to be very slow in our *in situ* TEM experiments, even the temperature is as high as 773 K, as partly shown in Figure 2b. The small crystalline size and the one-third fraction of the τ-phase formed via displacive mode can to some extent explain the higher coercivity and lower magnetization in MnAl powders obtained by flash-milling and post-annealing at lower temperatures [26].

## 4. Conclusions

A more complete understanding of the ε→τ PT in MnAl and MnAlC alloys has been developed based on the results of the current studies. The PT modes are mainly selected by temperatures. Only the displacive mode exists in the low temperature region, i.e., 510–650 K for MnAl and 540–766 K for MnAlC. The displacive mode and the massive mode coexist at higher temperatures, while the weight of massive mode increases with increasing temperature. Only one-third or less of the ε-phase can be transformed into τ-phase via the displacive mode and the remaining two-thirds or more via the massive mode. The typical growth rate of the τ-MnAlC grains at 773 K is 8–60 nm/s. High purity ferromagnetic τ-MnAl without any addition of stabilizers was prepared by controlling the temperature and heating time. Temperature (or time) dependence of magnetization measurements under magnetic fields is employed to determine the activation temperature (or PT rate) of both displacive and massive PT of ε→τ in MnAl-based magnets.

## Supplementary Materials

The following are available online at www.mdpi.com/1996-1944/10/9/1016/s1, Video 1: Massive growth of τ-phase from ε-MnAl(C).
